# Social hierarchies and meritocracy: objective status and the moderating role of subjective social status on perceived meritocracy

**DOI:** 10.3389/fsoc.2026.1750965

**Published:** 2026-06-19

**Authors:** Julio Iturra-Sanhueza, Juan C. Castillo, Juan Diego García-Castro

**Affiliations:** 1 Bremen International Graduate School of Social Sciences (BIGSSS), Bremen, Germany; 2 Universidad de Chile, Facultad de Ciencias Sociales, Departamento de Sociología, Santiago, Chile; 3 Centro de Estudios de Conflicto y Cohesion Social, Santiago, Chile; 4 Universidad de Costa Rica, Sede de Occidente, Costa Rica

**Keywords:** education, income, meritocracy, socioeconomic status, subjective social status

## Abstract

This study investigates how subjective and objective dimensions of social status shape perceptions of meritocracy within Chile’s highly unequal society. Drawing on a representative sample of the urban population from the 2018 wave of the Longitudinal Social Study of Chile (ELSOC; *N* = 3,488), this research examines whether subjective social status is associated with perceptions that effort and talent are rewarded, even after controlling for related factors such as income and education. Anchored in the *Reference Group and Reality (R&R) blend hypothesis*, the analyses provide evidence that subjective status is significantly and positively associated with perceived meritocracy. However, nuanced evidence is found for objective status. Income is often positively associated with perceived meritocracy, as individuals with greater economic resources may attribute their position to effort and talent (*self-interest hypothesis*). However, our results show a weak, statistically non-significant association for household income deciles, although alternative operationalizations show differences in meritocracy perception in the expected direction, albeit modestly. While education may reinforce meritocratic ideals through normative reinforcement (*socialization hypothesis*), it may also foster more critical views by increasing awareness of structural inequalities (*instruction hypothesis*). Our findings support the latter, as higher levels of education are associated with lower perceived meritocracy. Moreover, subjective status moderates the relationship between objective status indicators and perceived meritocracy. Individuals with higher income or educational attainment perceive greater meritocracy only when they also self-identify as higher in status. Conversely, those in advantaged positions but with low subjective status express lower perceived meritocracy. These findings highlight the importance of social comparison and self-perception in legitimizing or contesting meritocratic ideals, especially among upper-status groups. The study contributes to the literature on the link between social stratification and attitudes towards inequality by discussing the assumptions of rational self-interest and underscoring the psychosocial mechanisms underpinning status perception and perceptions of meritocracy. It also advances research beyond Western contexts by examining these dynamics in Chile, offering insights into how social hierarchies shape perceptions in highly unequal societies. This study shows that subjective social status is a relevant factor through which distributive outcomes are perceived and evaluated, underscoring that objective advantage alone does not guarantee belief in meritocracy. Limitations and directions for future research, particularly concerning social networks and status misperception, are also discussed.

## Introduction

Meritocracy is an ideal that promotes the distribution of resources in a society based on individual effort and ability (Young, 1961). In modern societies, merit is commonly embraced as a fair way to achieve status, rather than relying on social connections or inheritance (Hadjar, 2008). The attention of social scientists to the subjective aspects of meritocracy has increased over the past years, linked to rising economic inequality (Mijs, 2021; Piketty and Goldhammer, 2014) and its link with social mobility (Hertel and Groh-Samberg, 2019; OECD, 2018). Within this context, a primary focus of this research is how individuals perceive the functioning of meritocracy (Castillo et al., 2025; Mijs et al., 2022).

In recent years, studies on public attitudes toward economic inequality have examined whether subjective social status might displace the role of objective socioeconomic position. This might be the case because people are misinformed about their objective position (Cruces et al., 2013; Karadja et al., 2017; Melli and Azzollini, 2025). For instance, it has been shown that societies with high income inequality tend to be more biased, as a result of lower subjective status among middle- and top-income groups (Wiesner and Iturra-Sanhueza, 2025). These findings have motivated increased attention to the link between the subjective social status and its consequences for attitudes in the economic and political domains (Brown-Iannuzzi et al., 2015; Gidron and Hall, 2020; Kirsten et al., 2022).

There is robust evidence of a positive link between objective and subjective status, in which higher socioeconomic status positions are associated with higher subjective self-placement (Andersson and Lindh, 2023; Andersson, 2018; Castillo et al., 2013; Iturra and Mellado, 2018). One of the central claims of this literature is that perceptions of income inequality and stratification stem from a shared interaction of two elements: the material forces of an individual’s structural position and status perceptions derived from comparisons with reference groups (Evans et al., 1992; Evans and Kelley, 2017).

Regarding meritocracy, previous evidence has shown that individuals with higher status self-perception tend to perceive higher meritocracy (Castillo et al., 2018; Madeira et al., 2019). However, there is a knowledge gap regarding whether subjective social status can moderate the link between objective socioeconomic status and perceived meritocracy. Only a few studies have shown that subjective status serves as a moderating factor in general perceptions of inequality and in diagramatic perceptions of inequality, affecting redistributive preferences and meritocratic perceptions (Bobzien, 2019; Fatke, 2018). Nevertheless, to the best of our knowledge, less attention has been given to how subjective social status moderates the link between objective socioeconomic position and meritocracy perception.

Against this background, we expect status self-perception to be linked with perceptions on whether effort and talent are rewarded in society, core elements of the meritocratic ideal (Castillo et al., 2023). Thus, what is the role of subjective social status in the perception of meritocracy? Particularly, to what extent can subjective status moderate the association of objective position on perceived meritocracy? We argue that subjective social status represents a psychosocial process that explains why some people perceive lower (or higher) meritocracy even when they hold (dis)advantageous socioeconomic positions (Cruces et al., 2013; Wiesner and Iturra-Sanhueza, 2025).

The present study aims to examine the relationship between both objective and subjective social positions on perceptions of meritocracy. In terms of *objective* status, we focus on income and education as two distinctive socioeconomic status characteristics. From a social stratification perspective, income reflects individuals’ access to economic resources that support their material well-being and shape their chances of openly participating in social life (Sachweh, 2012; Wilkinson and Pickett, 2010). Beyond its material dimension, relative income position is considered a core indicator of self-interest, as individuals with a relative income position above the median are more opposed to redistributive measures (Meltzer and Richard, 1981). Education, in turn, captures the level of qualification that structures individuals’ labor market returns through occupational positions and wages (Connelly et al., 2016). Yet education also extends beyond economic advantages: higher levels of education are linked to more diverse cultural consumption and to the adoption of more libertarian or liberal socio-cultural values (Chan and Goldthorpe, 2007).

Regarding *subjective* social status, social comparison processes and reference groups play a crucial role in shaping how individuals perceive meritocracy as a psychosocial force. Inspired by the Reference Group and Reality Blend (R&R) approach (Evans, 2004; Evans et al., 1992), we hypothesize that material conditions—represented by objective socioeconomic status—and subjective perceptions of one’s position within the social hierarchy *jointly* influence perceived meritocracy in terms of how individuals in Chile perceive that rewards are distributed according to effort and talent (Young, 1961). In particular, our hypothesis posits that the positive link between status and perceived meritocracy is moderated by subjective social status. Based on the literature on the link between social status and perceptions of meritocracy (Canales, 2015; Castillo et al., 2018; Duru-Bellat and Tenret, 2012), we expect that individuals occupying higher socioeconomic positions and perceiving themselves as highly ranked will be more likely to perceive that merit in Chile is rewarded according to effort and talent.

### Meritocracy perception and objective social status

Merit is usually conceived as the combination of individual effort and ability (Sen, 2000). Moreover, the meritocratic ideal assumes that status achievement is not constrained by the structure of opportunities in society (McCall et al., 2017). Both constitute the substantive core of how meritocracy can be comprehended. However, studies on meritocracy have employed concepts such as meritocratic beliefs (Bernardo, 2021; Mijs, 2021) and perceived meritocracy (Duru-Bellat and Tenret, 2012) in an undifferentiated manner. This lack of conceptual and empirical accuracy is often evident in studies that address meritocracy’s subjective aspect without distinguishing between descriptive and normative dimensions (Castillo et al., 2018, 2023). Research on subjective meritocracy has focused on structural inequality as an explanatory factor in perceptions of meritocracy, primarily analyzing the role of social status, yielding mixed results due to differing measurement strategies for the concept.

In the distributive justice literature, perception is understood as the descriptive beliefs about empirical conditions (Janmaat, 2013). This descriptive dimension (*how is*) does not consider the normative evaluation of the object (*should be*) referred to as preferences (Janmaat, 2013). In this regard, Garcia (2001) suggests that perceived meritocracy is the degree to which individuals consider their society to comply with meritocratic principles. In other words, to what extent effort and talent are rewarded, indicating “how meritocracy is” rather than “how it should be”. Regarding this distinction, it has been suggested that perceptions are less stable than preferences across socioeconomic groups and require further studies to understand how these are formed (Castillo et al., 2018).

The link between income and perceived meritocracy shows that, in general, higher income is associated with greater perceived meritocracy (Castillo et al., 2018; Duru-Bellat and Tenret, 2012). According to the rational self-interest approach (Meltzer and Richard, 1981), higher-income individuals perceive higher meritocracy because they have accumulated economic resources through effort and talent (Morris et al., 2022). Comparative evidence shows that higher income goes in line with higher perceived meritocracy, either by the perceived importance of individual merit in the process of getting ahead in life (Duru-Bellat and Tenret, 2012; Reynolds and Xian, 2014) or the extent to which intelligence and skills are rewarded (Castillo et al., 2018; Duru-Bellat and Tenret, 2012). Nonetheless, there is mixed evidence on how lower-income individuals rationalize inequality and meritocracy. Some research supports the “activated conflict theory,” which suggests that people experiencing poverty in unequal localities hold lower perceptions of meritocracy due to social comparisons that increase awareness of social status (Newman et al., 2015). Further research, based on the “relative power theory,” challenges this finding, arguing that the rich hold more power due to income and wealth concentration. These advantages allow affluent groups to disseminate legitimizing ideas about economic inequality, as the poor are less critical of dominant beliefs, such as meritocracy, as a psychological mechanism for coping with deprivation (Solt et al., 2016). However, other research shows that low-income individuals in highly unequal contexts have firmer meritocracy beliefs, but the differences tend to fade as inequality declines (Morris et al., 2022).

Because income reflects an individual’s economic resources, social stratification scholars have highlighted the importance of educational attainment for status achievement (Connelly et al., 2016; Goldthorpe, 2003). Research on meritocracy perception has discussed two approaches, each with contested evidence regarding the role of educational attainment. On the one hand, the reproductionist approach (Bernstein, 2003; Bourdieu and Passeron, 2009) supports the *socialization* hypothesis, suggesting school socialization operates as a normative reinforcement of meritocratic ideals, institutionalizing social status inequalities and making educated individuals more willing to support meritocracy as a legitimizing mechanism (Lampert, 2013). On the other hand, the *instruction* hypothesis proposes that perceived meritocracy tends to weaken in individuals with higher educational attainment because they would be more aware of the influence of structural factors on status achievement (Duru-Bellat and Tenret, 2012), considering experiences of educational mobility (Canales, 2015) and higher chances to engage with complex political ideas (Bubak, 2019).

Evidence in favor of the socialization hypothesis has pointed out that education is associated with a higher importance of individual factors in getting ahead in life (Fatke, 2018; Mijs and Hoy, 2021; Reynolds and Xian, 2014), but also as a critical perceived determinant of wages (Kunovich and Slomczynski, 2007). Evidence supporting the *instruction* hypothesis, in contrast, shows that more educated individuals are less likely to believe that people are rewarded for their effort and talent (Castillo et al., 2018; Duru-Bellat and Tenret, 2012). The claims of the *instruction* hypothesis resonate with the idea that higher awareness of economic inequality may drive feelings of injustice and relative deprivation, as individuals with the same educational attainment may obtain unequal rewards (Adams, 1965; H. J. Smith et al., 2020). This has also been suggested as an explanation for why higher educational attainment is associated with greater perceived inequality between the rich and the poor, salary gaps, and diagrammatic inequality (Castillo, 2012).

### Subjective social status as a moderator between objective status and meritocracy

The study of perceived meritocracy has paid more attention to objective status or materialist explanations than to subjective self-perceptions of status (Canales, 2015; Kluegel and Smith, 1986). The anchoring of subjective social status in social comparison processes is another less-discussed social-psychological factor that might help explain perceptions of meritocracy alongside objective status (Castillo et al., 2022). The literature conceives subjective social status as the perception of an individual or group regarding their own standing in the social hierarchy relative to a reference group, such as top-bottom groups in society (Evans, 2004; Kunovich and Slomczynski, 2007) or their neighbors and local community (Condon and Wichowsky, 2020). An understudied topic is whether subjective social status can moderate the link between socioeconomic status and perceived meritocracy. There is evidence on the misalignment between objective and subjective status (Cruces et al., 2013; Wiesner and Iturra-Sanhueza, 2025). However, only a few studies have examined how subjective social status can moderate attitudes toward inequality (Bobzien, 2019; Fatke, 2018).

Previous contributions in the social comparison literature have argued that individuals prefer to compare themselves with people or groups perceived as similar, in which these reference groups serve to reduce cognitive dissonance and help them form better estimates of their own attributes (Festinger, 1954; Merton and Rossi, 1968). In the sociological literature, the Reference Group and Reality blend (R&R) approach (Evans et al., 1992; Kelley and Evans, 1995) suggests that status perception can be explained by the characteristics of the reference groups involved in the comparison. Accordingly, perceptual biases may be explained by an availability heuristic that serves as a cognitive dissonance-reduction mechanism in situations with limited information (Evans et al., 1992; Kelley and Evans, 1995). Reference group homophily may reinforce their imagined ideas about the distribution of economic resources and status positions in society. Therefore, it is arguable that subjective social status is influenced by both the material forces of socioeconomic position and the characteristics of the social environment where an individual is embedded (Kim and Lee, 2021; Mayerhoffer and Schulz, 2022; Mijs and Usmani, 2024; Son et al., 2021).

Empirically, among the most relevant socioeconomic factors that explain subjective social status, a positive link has been found between education, occupational status, and income (Andersson and Lindh, 2023; Castillo et al., 2013; Marqués-Perales and de la Rodríguez- Fuente, 2023). The discrepancies between objective and subjective status are linked to the persistent “middle-class” bias observed across various societies, in which low-status individuals tend to overestimate their position while high-status individuals underestimate it (Andersson, 2018; Castillo et al., 2013; Lindemann and Saar, 2014). These biases have been discussed in the literature as a possible explanation of why the self-interest model fails to predict the link between relative income position and redistributive preferences (Cansunar, 2021; Cruces et al., 2013). The central claim is that this literature holds that reference groups affect subjective estimates of inequality; therefore, subjective social status can lead to outcomes different from those associated with objective status characteristics (Schneider, 2019).

Building on the stated literature, it is plausible to consider subjective status not only in terms of direct association but also as a moderator of the link between objective socioeconomic status and perceived meritocracy. Related evidence on the direct influence of subjective status shows that those who perceive themselves in higher social positions tend to hold stronger legitimizing views of economic inequality through system justification (Vargas-Salfate et al., 2018), internal poverty attribution, and the justification of salary gaps (Schneider and Castillo, 2015). In Chile, a study found that higher subjective status is associated with greater perceived rewards for effort and intelligence (Castillo et al., 2018).

Studies on subjective social status as a moderating factor have primarily focused on its relationship with other subjective variables rather than on its interaction with socioeconomic status. It has been shown that individuals with high subjective social status and a high perception of meritocracy tend to justify income inequality to a greater extent (Bernardo, 2021). Related evidence has shown that the positive association between perceived inequality and support for redistribution becomes weaker as subjective status increases (Bobzien, 2019). Regarding meritocracy, another study shows that the negative association between perceived economic inequality and perceptions of meritocracy becomes weaker among those with higher subjective social status (Fatke, 2018).

Experimental evidence using informational priming shows that correcting positional bias affects the income–attitude relationship predicted by self-interest. When individuals who previously overestimated their social status learn their objective position, they tend to increase their support for redistribution (Cruces et al., 2013). Highly educated individuals who have more accurate perceptions of their subjective position and stronger meritocratic beliefs tend to be less responsive to informational manipulations, as their views are already more closely aligned with their economic interests (Karadja et al., 2017). Regarding status misperception, research shows that when individuals are informed about their actual position in the income distribution, those with biased estimates—particularly those in the lowest and highest income strata—tend to perceive greater inequality (Hvidberg et al., 2020).

Against this background, while some evidence does not directly address perceived meritocracy, it offers empirical insights into how subjective social status might nuance the relationship between socioeconomic position and perceived meritocracy. Therefore, based on the above theoretical and empirical insights, it is plausible to empirically examine whether the relationship between income and education in perceived meritocracy is moderated by subjective social status.

## Current study

**Figure 1:**
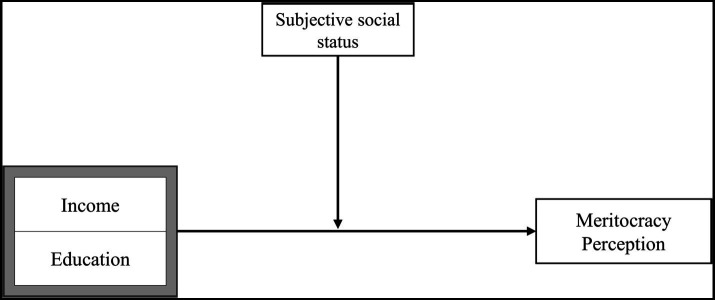
Theoretical model.

To summarize our hypotheses, Figure 1 depicts the theoretical model proposed in the study.

Based on the literature (Castillo et al., 2018; Duru-Bellat and Tenret, 2012; Morris et al., 2022; Newman et al., 2015; Solt et al., 2016), we expect that individuals in higher-income positions will perceive greater meritocracy, as they have experienced greater economic returns in the labor market (*self-interest*). In addition to rational economic motivation, we also expect educational attainment to be linked to higher perceived meritocracy, driven by normative reinforcement from educational achievement (*socialization*). Nevertheless, we also recognize the possibility that the hypothesized association between educational attainment and the development of more critical views towards meritocracy may coexist. The direct implication is that, especially among the highly educated, who usually consider effort and talent important factors for getting ahead in life, they may also hold a critical view of the current distribution of rewards according to merit (*instruction*).

Thus, we expect that individuals with higher objective status will perceive that rewards in society are distributed according to effort and talent. However, we recognize the competing alternative explanation for education presented above.

1. Objective social status [income (H1a) and education (H1b)] is positively related to perceived meritocracy.
2. Alternatively, higher educational attainment is negatively related to perceived meritocracy (H1c).

Subjective social status is reflected in individuals’ self-placement based on their experience with economic inequality, as reflected in comparisons with their reference groups (Bobzien, 2019; Cruces et al., 2013; Fatke, 2018; Karadja et al., 2017). When objective and subjective status are aligned at a high level—for example, a high-income individual who also perceives themselves as having high social standing—we expect higher perceived meritocracy. Conversely, a scenario in which someone has a high income but a lower subjective status can become disruptive, leading to questioning the meritocratic nature of their position. Accordingly, we hypothesize that:

1. Subjective social status moderates the relationship between objective status [income (H2a) and education (H2b)] and perceived meritocracy.

To test these hypotheses, we use data from the wave 2018 of the Chilean Longitudinal Social Survey (ELSOC). We build on previous studies of Chile that have described it as an “adjudicative case” for studying phenomena related to social and economic inequality. Recent estimates show that in 2017, the top 1% and top 10% of households in Chile accounted for 17.3% and 49.1% of disposable income, respectively (López Moreno et al., 2026). Past studies on intergenerational social mobility in Chile have evidenced that the class structure is far from being impermeable across class boundaries. However, permeability from lower to lower-middle positions has been more common than in the more selective and socially enclosed upper-middle classes (Torche, 2005), a pattern further confirmed by income inequality between the upper-middle and the rest of the social classes (Espinoza and Núñez, 2014). This pattern has also been confirmed in comparative research, where Chile displays “particularly high levels of between-class inequality combined with low levels of social fluidity” (Hertel and Groh-Samberg, 2019, p. 14). Given these reasons, the Chilean context stands as a relevant context to scrutinize the above hypothesis on the relationship between social status characteristics and meritocracy.

Against this background, the present article makes two contributions to the literature. On the one hand, it tests the relationship between objective status (education and income) and perceived meritocracy in a non-Western, Educated, Industrialized, Democratic, or Rich (non-WEIRD) country, a relationship that has yielded divergent results in the literature (Castillo et al., 2022; Duru-Bellat and Tenret, 2012; Kunovich and Slomczynski, 2007; Newman et al., 2015). On the other hand, extending previous research (e.g., Bobzien, 2019; Fatke, 2018), we directly test the expected moderating effect of subjective status on the relationship between objective socioeconomic status and perceived meritocracy.

### Data, variables, and methods

#### Data

The data used is a representative sample of the urban population of Chile, corresponding to the third wave of the Longitudinal Social Study of Chile (COES, 2018), an annual study conducted by the Center for the Study of Conflict and Social Cohesion (COES). After randomly selecting 1,067 blocks, 3,748 men and women aged 18 to 75 from 94 communes across the country were selected to participate in face-to-face interviews. The respondents represented 93% of urban dwellers and 77% of Chile’s population. The overall response rate for the survey was 69% (see ELSOC Methodological Manual for details). After the listwise deletion of missing values, the analytical sample comprises 3,488 individuals.^1^

#### Variables

The dependent variable is perceived meritocracy, employing two indicators. First, “In Chile, people are rewarded for their efforts” as the dimension of effort, and second, “In Chile, people are rewarded for their intelligence and skills” as the dimension of talent. Both indicators are five-category Likert scales, with higher values indicating stronger perceptions of meritocracy, and they are strongly correlated (*r* = 0.76). Then, following previous comparative studies, we compute an average score of the two indicators (*M* = 2.8, *SD* = 0.9) to capture the combined degree of agreement on whether effort and intelligence are rewarded in Chile.

Subjective social status is measured using an 11-point scale variable in which respondents identify themselves on the social ladder by answering, “*In our society, some groups are usually at the higher levels, and others tend to be placed at the lower levels of society. Using the presented scale, where 0 is the lowest level, and 10 is the highest level, where would you put yourself in Chilean society?*” where higher values represent a higher perceived social status (*M* = 4.4, *SD* = 1.6).

The objective status position is measured through three variables. First, the *Household Income Decile* is calculated using monthly per capita household income and categorized into deciles. Second, *Educational level* corresponds to the five categories for educational attainment following the International Standard Classification of Education (UNESCO, 2012). In addition, gender (Female = 62%) and age (*M =* 47*, SD =* 15) are included as controls (see Supplementary Table S7).

## Methods

Considering the dependent variable as a continuous, stepwise ordinary least squares regression model is used (Wooldridge, 2013). First, models are estimated for each independent variable. Second, a series of estimated models considers the interaction between objective position and subjective status. Finally, interaction effects are presented using conditional linear predicted values.

## Results

### Descriptive results on perceived meritocracy, subjective and objective status

**Figure 2:**
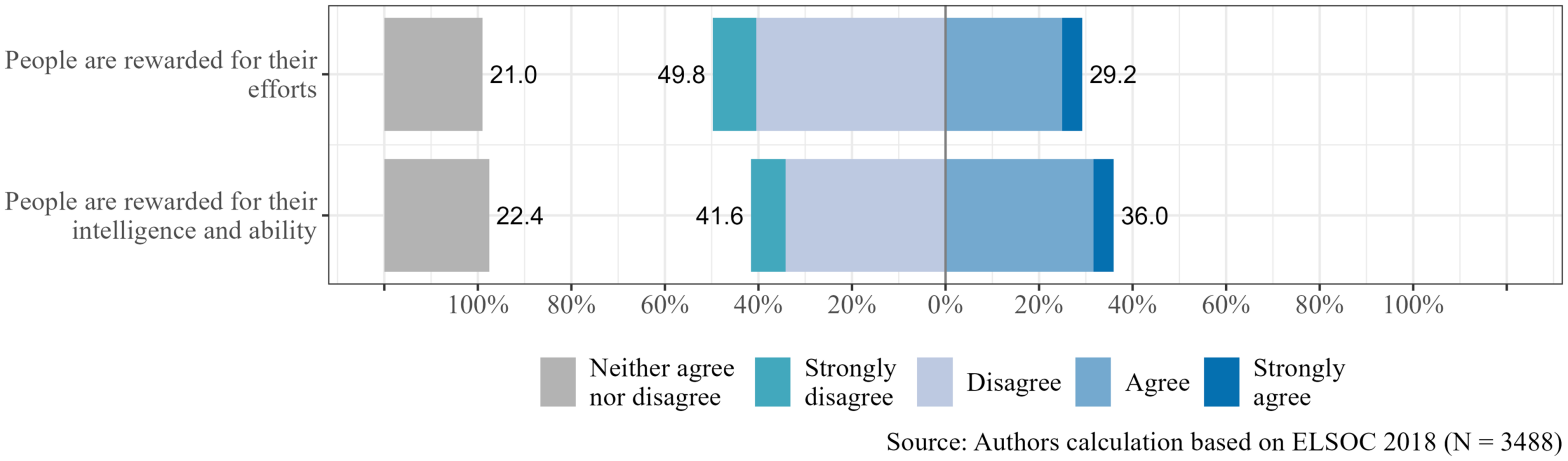
Distribution of responses in the perceived meritocracy items.

Figure 2 depicts the distribution of responses to the two indicators of meritocracy, showing that most individuals tend to disagree or strongly disagree. For example, regarding the item about how *effort* is rewarded, 49.8% answered that they disagree or strongly disagree. Similarly, the perception regarding rewards for *intelligence and ability* shows a decrease in disagreement to 41.6%. Finally, about 22% of respondents say they neither agree nor disagree with the idea that talent and effort are rewarded in Chile.

**Figure 3:**
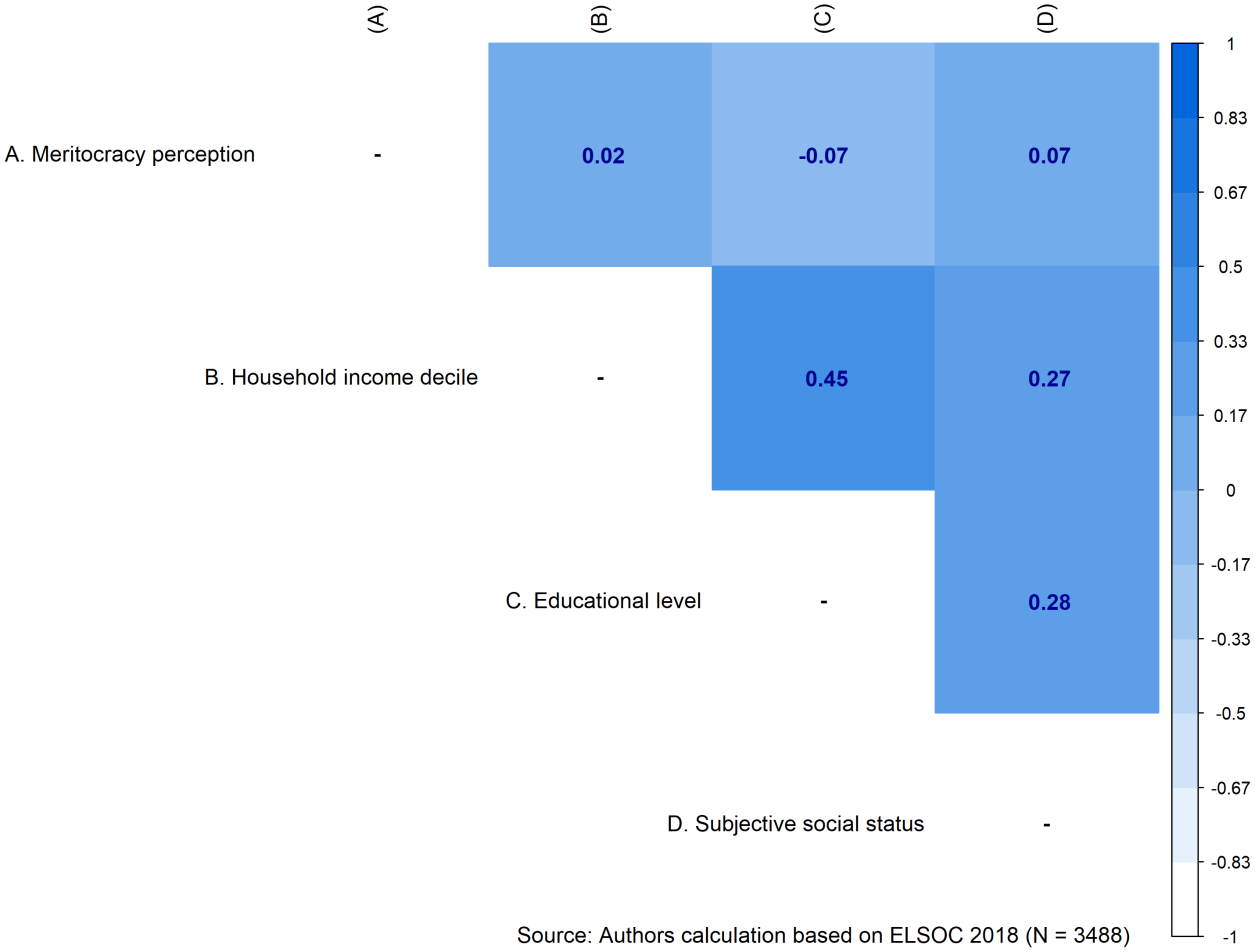
Bivariate associations of perceived meritocracy, household income, education, and subjective social status.

Figure 3 shows that perceived meritocracy and subjective status have a weak positive correlation (*r* = 0.02, *p* < 0.01). On the side of objective status, perceived meritocracy has a weak positive correlation with income decile (*r* = 0.06, *p* < 0.01), suggesting that higher economic resources are associated with stronger perceived meritocracy in line with our hypothesis 1a. In contrast, educational level shows a weak negative correlation with perceived meritocracy (*r* = 0.07, *p* < 0.01), suggesting that more educated individuals hold more critical views of meritocracy, contrary to our main hypothesis 1b, but in the direction predicted by the alternative hypothesis 1c.

### The relationship between objective and subjective status on meritocracy perception

Model 1 in Table 1 shows that household income has a positive but insignificant association (*β* = 0.001, *p* > 0.05), therefore not supporting the rational self-interest hypothesis (H1a). The results in Model 2 show that as educational level increases, perceptions of meritocracy decrease, providing evidence in favor of the alternative instruction hypothesis (H1c) rather than the main socialization hypothesis (H1b). This suggests that people with higher education tend to view meritocracy more critically, with this view consistently higher across all rungs of the educational ladder. For instance, the results indicated that participants with primary or lower secondary education have significantly lower meritocracy perceptions compared to those with primary or less education (*β* = −0.18, *p* < 0.05). Similarly, upper secondary (*β* = −0.20, *p* < 0.001), short cycle tertiary (*β* = −0.30, *p* < 0.001), and tertiary or higher (*β* = −0.33, *p* < 0.001) educational levels also had a significantly lower perceived meritocracy compared to the less educated group. As expected, Model 3 showed that those who perceived themselves higher in the social ladder also perceived more meritocracy in society (*β* = 0.05, *p* < 0.001).

**Table 1:** Linear regression for socioeconomic status, subjective social status perception of meritocracy.

Predictors	Model 1	Model 2	Model 3	Model 4	Model 5
Income Decile	0.001		0.006	−0.070^***^	0.004
	(0.006)		(0.007)	(0.018)	(0.007)
Education (ref: Incomplete Primary or less)					
Primary and Lower secondary		−0.194^*^	−0.200^**^	−0.188^*^	−0.566^**^
		(0.078)	(0.077)	(0.077)	(0.200)
Upper secondary		−0.207^***^	−0.227^***^	−0.216^***^	−0.688^***^
		(0.060)	(0.061)	(0.061)	(0.157)
Short-cycle tertiary		−0.312^***^	−0.352^***^	−0.345^***^	−0.861^***^
		(0.067)	(0.072)	(0.071)	(0.199)
Tertiary or higher		−0.340^***^	−0.422^***^	−0.443^***^	−0.996^***^
		(0.063)	(0.073)	(0.073)	(0.183)
Subjective Social Status (SSS)			0.053^***^	−0.037	−0.052
			(0.011)	(0.023)	(0.031)
Income × SSS				0.016^***^	
				(0.004)	
Primary and Lower secondary × SSS					0.093^*^
					(0.046)
Upper secondary × SSS					0.116^**^
					(0.036)
Short-cycle tertiary × SSS					0.126^**^
					(0.043)
Tertiary or higher × SSS					0.137^***^
					(0.038)
Female (ref. = Male)			0.166^***^	0.172^***^	0.166^***^
			(0.033)	(0.033)	(0.033)
Age			0.002	0.002	0.002
			(0.001)	(0.001)	(0.001)
R^2^	0.000	0.010	0.026	0.031	0.030
Adj. R^2^	−0.000	0.009	0.023	0.029	0.026
Num. obs.	3,488	3,488	3,488	3,488	3,488

^***^p < 0.001; ^**^p < 0.01; ^*^p < 0.05. Standard errors in parentheses.

**Figure 4:**
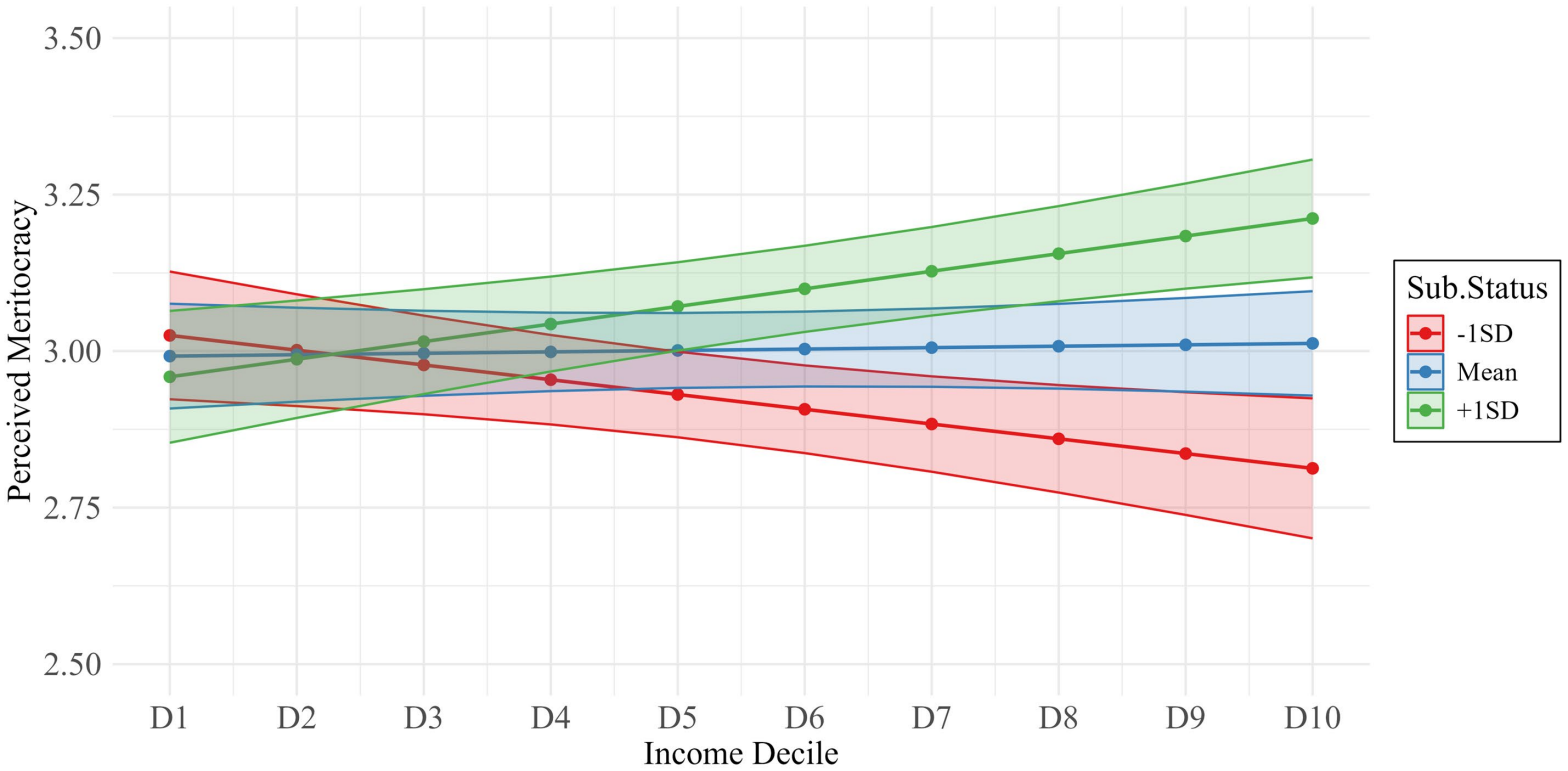
Interaction of household income and subjective social status on meritocracy perception.

Model 4 shows that subjective social status positively moderates the association of household income with perceived meritocracy (*β* = 0.02, *p* < 0.001). Compared to the direct association between household income and meritocracy, here we observe differences in perceived meritocracy depending on how individuals’ subjective social status. For example, we see that subjective self-placement does not differ for people in the bottom 50% of the income distribution (D1 to D5). Still, it does show differences for those above the median, up to the top 10% most affluent (D6 to D10). Figure 4 shows that for those with one standard deviation above the mean on the subjective status scale, being in the 10th income decile predicts a perceived meritocracy score of 3.2 (95% CI [3.1, 3.3]). In contrast, for those with one standard deviation below the mean in subjective status, being in the highest income decile yields a predicted value of 2.8 (95% CI [2.7, 2.9]) on the perceived meritocracy scale. In other words, our evidence suggests that for individuals at the middle and top rungs of the income distribution, their subjective social status can widen the perceived meritocracy divide, both for those with low and high subjective social status.

**Figure 5:**
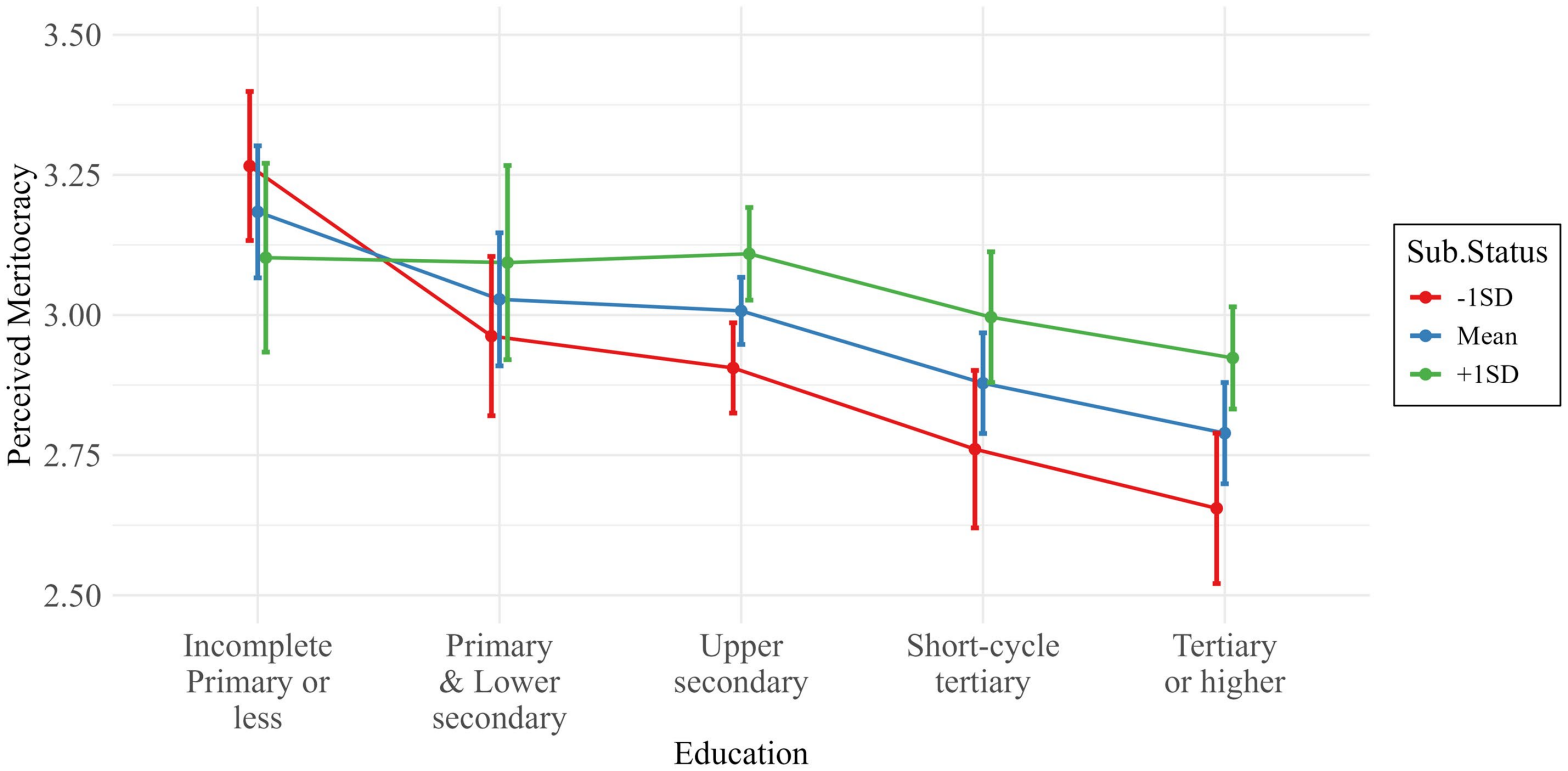
Interaction of education and subjective social status on meritocracy perception.

Model 5 shows that subjective social status positively moderates the negative association between educational level and perceived meritocracy, particularly among short-cycle tertiary (*β* = 0.13, *p* < 0.001) and tertiary or higher (*β* = 0.14, *p* < 0.001). To better illustrate these results, Figure 5 depicts the predicted mean values of perceived meritocracy. First, the graph shows that subjective status does not moderate perceived meritocracy among those with secondary education or less. For instance, for participants with incomplete primary education, the predicted perceived meritocracy was 3.2 (95% CI [3.1, 3.4]) when subjective social status was one standard deviation above the mean, and 3.1 (95% CI [2.8, 3.0]) when it was one standard deviation below the mean. In contrast, among those with a tertiary or higher educational degree, subjective social status moderates perceptions of meritocracy. For this group, the predicted perceived meritocracy was 2.6 (95% CI [2.5, 2.7]) when subjective social status was one standard deviation over the mean, and 3.1 (95% CI [2.8, 3.0]) when it was one standard deviation below the mean. Therefore, despite this group having the most critical perceptions of meritocracy—similar to what was observed in the interaction between household income deciles—those with higher educational credentials tend to hold different views about how meritocracy operates in society. This would be in part explained by subjective status among the most educated.

### Robustness analysis

First, given the null evidence on the linear association between household income decile and meritocracy perception, we also consider two alternative income operationalizations (see Supplementary Tables S1, S2). One alternative is using the Top 10% (Decile 1), Middle 50% (Deciles 5 to 9), and the Bottom 40% (Deciles 1 to 4). The results show that the Top 10% has a positive, significant difference with both the Middle 50% and the Bottom 40%. However, the difference between the Middle 50% and the Bottom 40% are not statistically significant. A second alternative is based on the ratio between the individual household income and the median household income resulting in four relative income groups: poverty (<50 per cent of median income), below average (> = 50 per cent and <100 per cent), above average (> = 100 per cent and <200 per cent), and affluence (>200 per cent). The results indicate that the affluent show positive, statistically significant differences compared with the poverty and above-average groups. However, no statistically significant differences between the above- and below-average groups, nor between the latter groups and those in poverty. The interaction effects for both alternative income operationalizations replicate the original findings for income decile (see Supplementary Table S1).

Second, we estimated ordinal logistic regression models for each individual indicator (effort and talent) of the perceived meritocracy index (see Supplementary Table S3). Here, the results replicate the main findings reported in Table 1. Worth noticing that formal tests indicated violations of the proportional odds assumption for several predictors (see Supplementary Table S4).

Third, we tested an alternative operationalization of education, focusing on those with university degrees or higher compared with the rest of the educational groups. We find that those with a college degree perceive society as less meritocratic than those with a short-cycle tertiary education or lower (see Supplementary Table S5). The interaction effect with subjective social status shows that higher subjective social status negatively moderates the association between university education and perceptions of meritocracy. For those with a university degree compared with those with short-cycle education or lower, an increase in subjective social status is associated with a higher perception of meritocracy.

Fourth, we included migration status to compare immigrants with native-born individuals, as well as ethnic group membership versus non-membership. People with a migrant background perceive society as more meritocratic than non-migrants, with a statistically significant difference. In addition, members of indigenous communities perceive society as less meritocratic than those who are not. However, these differences are not statistically significant (see Supplementary Table S6).

## Discussion

This study shows that when individuals perceive themselves as being closer to groups at the top of society, they tend to agree that effort and talent are rewarded. These findings are aligned with previous research on individualistic poverty attributions (Schneider and Castillo, 2015) and perceived meritocracy (Castillo et al., 2018). In general, the results align with the self-interest hypothesis’s prediction regarding subjective self-placement. However, about objective socioeconomic status, our measure of income position based on household income deciles is not associated with perceived meritocracy, while it decreases with education. In both cases, the association of subjective social status with perceived meritocracy remains robust. These results align with our theorization that subjective social status is positively associated with perceptions of meritocracy. As previous evidence suggests, groups at the top of the social hierarchy are usually viewed as having high wages, well-educated and prestigious occupations, and are often seen as deserving winners; thus, higher subjective social status is also linked to higher perceived meritocracy (Roex et al., 2018).

The results do not fully support the economic self-interest hypothesis based on income decile (H1a), which predicts that material factors, such as household income, would significantly differentiate individuals’ perceptions of meritocracy. In this regard, we find no significant association with household income decile and perceived meritocracy. However, it should be noted that our findings do not imply that there are no differences between income groups; these differences are rather small when comparing income deciles and are sensitive to the measurement strategy for income positions, as noted in the robustness analysis. It may be that a within-group social comparison effect explains the positive but limited link between income and meritocracy. Previous evidence shows that the income-meritocracy link weakens when controlling for income differences within educational groups (Castillo et al., 2018). As previous studies show, a possible explanation is that the gap in meritocracy beliefs between high- and low-income groups narrows when income inequality is high (Morris et al., 2022; Solt et al., 2016). This leads us to conclude that, given our evidence, the support for the claim of a positive association between income and meritocracy goes in the expected direction but is rather weak.

Regarding education, our results contradict the idea that experiences in the educational system can foster meritocratic perceptions (Lampert, 2013) by internalizing meritocratic norms through *socialization* in academic institutions (H1b). As Duru-Bellat and Tenret (2012) stated, our findings show that higher levels of education may lead to more critical views of the distributive system and the idea that effort and talent are rewarded in society, rather than reinforcing meritocratic ideals. This also echoes previous studies in Chile showing higher perceived inequality and meritocracy among highly educated individuals (Castillo et al., 2012, 2018). This evidence is rather supportive of the competing hypothesis of *instruction* (H1c) that posits lower meritocracy perception among the highly educated. This could be partially explained by the fact that education, particularly tertiary education, is linked to greater contact with diverse socioeconomic groups, which, in turn, contribute to social learning about the life conditions of others in economic despair (Londoño-Vélez, 2022). Related evidence shows that perceived meritocracy can decrease, especially among the upper-middle class, when socioeconomic diversity among contacts increases (Otero and Mendoza, 2023). In an unequal context like Chile, experiences of social mobility may not align with expected economic returns (Espinoza and Núñez, 2014), which, in turn, is reflected in lower perceived meritocracy among highly educated groups (Canales, 2015).

To scrutinize an expectation inspired by the theoretical claims of the Reference Group and Reality blend (R&R) hypothesis, we tested the moderating role of subjective status on objective reality. First, the results align with previous evidence on the role of subjective social status on perceived diagrammatic inequality (Bobzien, 2019) or perceived income inequality (Fatke, 2018). Second, we found that the conditional effect of status on perceived meritocracy is especially salient as socioeconomic status increases. Our findings align with previous research on how status perception can lead to attitudinal change across different status groups (Cruces et al., 2013; Hvidberg et al., 2020). As expected, individuals in top-income positions and those who see themselves in higher-status positions tend to perceive higher levels of meritocracy. In contrast, those with higher income and education but lower subjective status tend to be more skeptical of meritocracy. In educational groups, differences in subjective social status create a divide, especially among those with tertiary education or higher.

A possible theoretical explanation of the association between education and meritocracy is that social comparison, driven by limited information arising from homophily in social networks, may lead to biased perceptions (Kim and Lee, 2021). Nevertheless, this assumption cannot be directly tested with the current data, as it would require information on the level of socioeconomic homogeneity within social environments (Iturra-Sanhueza, 2025). Another possible theoretical explanation for those with high socioeconomic status but low subjective social status is that social comparison processes may lead to subjective experiences of deprivation (Schneider, 2019), even among those who are objectively at the top of the social hierarchy. As proposed by Condon and Wichowsky (2020), upward comparisons may create anxiety among individuals across all status groups, leading to deprivation, lower subjective status, and an increased demand for redistribution. Also, feelings of being under-rewarded can lead to a sense of relative deprivation linked to expected returns (Adams, 1965). In the context of meritocracy, merit can be understood as a representation of the efforts and talent invested in educational attainment. Thus, we expected that those who perceive themselves as lower in the social hierarchy would also perceive lower levels of meritocracy, especially in positions that are affluent, highly educated, and skilled. While these groups may be objectively in high socioeconomic positions, some may still perceive themselves as belonging to the lower social strata, triggering a critical evaluation of meritocracy.

The moderating role of subjective status, particularly among highly educated individuals, may reflect a context in which economic opportunities do not meet expectations fostered by educational attainment (Canales, 2015; Espinoza and Núñez, 2014). Another plausible, and somewhat contrasting, explanation is that highly educated individuals from working-class backgrounds or with lower-educated parents may retain a relatively lower subjective social status despite experiencing upward mobility. In this case, their self-perceptions may remain anchored in their origins rather than fully adjusting to their achieved position. Moreover, their continued embeddedness in socially heterogeneous networks—spanning both origin and destination groups—may expose them to diverse experiences of advantage and disadvantage. This cross-class exposure can attenuate strictly meritocratic interpretations of success, fostering a more structural understanding of inequality. In line with research showing that subjective mobility and social context shape meritocratic beliefs, individuals with mixed social ties may be less inclined to attribute outcomes solely to effort or talent, and more likely to recognize the role of structural constraints. When educated individuals do not perceive their status as matching their credentials, it may lead to more critical views of meritocracy (Mijs et al., 2022). This relationship, which might not be exclusive to Chile, raises questions in a comparative perspective. For instance, as income inequality reduces positioning bias by enhancing subjective social status across all status groups (Wiesner and Iturra-Sanhueza, 2025; Schneider, 2019), one question that remains to be answered is whether the role of subjective status in perceived meritocracy varies in countries with different levels of income inequality and economic prosperity.

In line with the empirical distributive justice literature, we conceptualize subjective social status within the frameworks of social comparison processes and relative deprivation, which hold that subjective self-evaluations of status are key to explaining subjective well-being (Schneider, 2019). Here, we extend this argument to the perception of meritocracy and particularly as a moderator of income and education as measures of objective socioeconomic position. However, other interpretations might also be plausible, but are beyond the scope of this research. Another conceptualization relies on normative orientation. It has been argued that subjective social status reflects the extent to which individuals feel integrated into society. Gidron and Hall (2020) argue “that participation in the normative order depends on processes of social interaction through which people acquire acceptance as peers” (p.4). Thus, subjective social status might also reflect whether individuals feel socially marginalized or integrated. It is reasonable to conceive that subjective social status might operate as a mediating psychological process. Evidence on the link between inequality and well-being shows that part of the negative effect of income inequality is partially mediated by subjective social status, arguing that higher inequality increases the distance between income groups, which in turn anchors social comparisons with upper-income groups rather than the average income (Schneider, 2019). Following that rationale, this could also hold for perceived meritocracy.

The Chilean case provides important contextual leverage for interpreting these findings. In a setting characterized by high inequality and pronounced social segmentation, the persistent association between subjective social status and perceived meritocracy underscores the relevance of social comparison processes in shaping evaluations of fairness, beyond individuals’ objective position. At the same time, the weak and non-linear differences across income groups—particularly the absence of a clear gradient when using income deciles—suggest that the link between economic resources and meritocratic beliefs is more limited and sensitive to measurement than standard self-interest accounts would predict (Meltzer and Richard, 1981). This is especially notable in a highly unequal context, where clearer attitudinal divides might be expected, yet where prior research indicates that higher-status groups can also express relatively critical views of inequality (Dimick et al., 2017; Sachweh and Sthamer, 2019). In addition, the negative association between educational attainment and perceived meritocracy challenges the expectation that more educated and skilled individuals hold attitudes aligned with material self-interest. Taken together, these patterns highlight how the Chilean context helps reveal the limits of purely objective explanations and the importance of incorporating subjective status into analyses of meritocratic beliefs.

## Conclusion

This study is not without limitations. To measure meritocratic perception, we conceptualized it as two core dimensions of merit: effort and talent. We also recognize that in the current literature, other aspects of meritocracy can be addressed, such as non-meritocratic perceptions referred to family inheritance or social connections (Castillo et al., 2023). Regarding the explained variance in our models, this is rather low, with around 3% at best. Nevertheless, in the context of social science research, it is acceptable as long as there is no evidence of multicollinearity and the predictors included in the estimations are substantially connected to the theory, and the derived hypothesis aims to falsify (Ozili, 2023; Kohler et al., 2024). Scholarly research on meritocracy beliefs has shown that, when it comes to explaining the variance of individual characteristics, this tends to be rather low. For example, previous evidence shows that individual characteristics explained 4% of the variance in meritocratic beliefs in the United States of America, compared with 2% in China and Chile (Reynolds and Xian, 2014; Otero and Mendoza, 2023). Additionally, it has been shown that, in addition to individual factors, local contexts within countries, such as income inequality, also explain perceptions of meritocracy (Morris et al., 2022; Solt et al., 2016). Also, recent evidence suggests that the claimed bias between objective and subjective measures of status might be explained by different interpretations of “top-bottom” rungs and argues for revising how reference groups are operationalized to consider the multidimensionality of socioeconomic position (Anderson and Aussant, 2025). In terms of the research design, we exercise caution when interpreting our evidence, as the correlational nature of the data does not allow us to draw causal claims.

Another area of future research is considering the role of social networks in understanding the formation of status self-perceptions. This is especially the case in segregated networks with highly homogeneous same-status ties, which provide limited information on others’ living conditions that might affect perceptions of income distribution and status positions (Schulz et al., 2022). Considering our findings and previous studies, the intermediate and affluent income groups seem to be more responsive, either to concerns about procedural justice or to the negative externalities of income inequality (Dimick et al., 2017; Sachweh and Sthamer, 2019). Extending this to the micro-level, if we consider lower subjective status as an indicator of relative deprivation (Schneider, 2019), we might also expect higher support for redistribution among the relatively affluent.

Although the ELSOC is a longitudinal survey, we have taken an initial step, primarily because we need clarity on whether the hypothesized associations hold in a cross-sectional context. Considering this, our results raise longitudinal questions, such as the role of intergenerational and intragenerational educational mobility in subjective self-placement and attitudes toward inequality. To this end, future research should take a step forward and theorize on whether changes in structural positions also affect other attitudes related to stratification and economic inequality in Chile.

In conclusion, expanding on previous literature (e.g., Bobzien, 2019; Fatke, 2018), this study underscores that an individual’s subjective sense of their place in the social hierarchy is not merely a reflection of their objective position but a powerful psychosocial lens through which the fairness of the social system is evaluated. Our finding that subjective social status moderates the link between objective socioeconomic status and perceived meritocracy reveals that high income or education alone does not guarantee a perception of higher meritocracy; such perception is strongest when a corresponding perception of high social standing matches high objective positions. This highlights the role of social comparison and self-perception as fundamental mechanisms in legitimizing—or contesting—meritocratic ideals, particularly among high earners and the most educated in highly unequal societies.

## Data Availability

Publicly available datasets were analyzed in this study. This data can be found at: Reproducible Research, Centre for Social Conflict and Cohesion Studies COES, 2020, “Estudio Longitudinal Social de Chile 2018,” doi: 10.7910/DVN/H8OVMF, Harvard Dataverse, V2, UNF:6:nO/9Gb/t6CAdvLlY4/03tA== [fileUNF].

## Footnotes

1 In the following link, data and analysis code are available: https://osf.io/7gacz/overview?view_only=dd769c01d3fe406d86be52a80f88cf27
