# Supplementary material for: Social hierarchies and meritocracy: objective status and the moderating role of subjective social status on perceived meritocracy
Source: Front Sociol. 2026 Jun 19;11:1750965. doi: 10.3389/fsoc.2026.1750965 (PMC13330480; doi:10.3389/fsoc.2026.1750965)
Supplement: Supplementary file 1 [file Data_Sheet_1.PDF]

SUPPLEMENTARY ONLINE MATERIALS

for

Social hierarchies and meritocracy: objective status and the moderating role of subjective social status on perceived meritocracy

Frontiers in Sociology

Version: 2026.

Supplementary materials

Table S1: Linear Regression for socioeconomic status, subjective social status perception of meritocracy for different income measures

	Model 1	Model 2	Model 3	Model 4
Intercept	2.771 (0.097)***	2.905 (0.110)***	2.752 (0.100)***	2.959 (0.137)***
Income: Top-Bottom (ref.= Bottom 40%)				
Middle 50%	-0.042 (0.037)	-0.234 (0.109)*		
Top 10%	0.124 (0.063)*	-0.601 (0.222)**		
Middle 50% × SSS		0.045 (0.024)		
Top 10% × SSS		0.135 (0.039)***		
Income: Median share (ref.= Poverty)				
Below average			0.046 (0.050)	-0.061 (0.143)
Above average			-0.031 (0.052)	-0.361 (0.155)*
Affluence			0.142 (0.060)*	-0.368 (0.174)*
Below average × SSS				0.026 (0.032)
Above average × SSS				0.078 (0.034)*
Affluence × SSS				0.109 (0.035)**
Education (ref.= Incomplete Primary or less)				
Primary & Lower secondary	-0.197 (0.077)*	-0.189 (0.077)*	-0.208 (0.077)**	-0.197 (0.077)*
Upper secondary	-0.219 (0.061)***	-0.209 (0.061)***	-0.231 (0.061)***	-0.222 (0.061)***
Short-cycle tertiary	-0.345 (0.071)***	-0.332 (0.071)***	-0.368 (0.071)***	-0.361 (0.071)***
Tertiary or higher	-0.426 (0.072)***	-0.430 (0.072)***	-0.444 (0.072)***	-0.458 (0.072)***
Subjective Social Status (SSS)	0.049 (0.011)***	0.014 (0.017)	0.050 (0.011)***	-0.002 (0.026)
Female (ref.= Male)	0.174 (0.033)***	0.176 (0.033)***	0.167 (0.033)***	0.169 (0.033)***
Age	0.002 (0.001)	0.002 (0.001)	0.002 (0.001)	0.002 (0.001)
R^2	0.028	0.031	0.029	0.033
Adj. R^2	0.025	0.028	0.026	0.029
Num. obs.	3488	3488	3488	3488

***p < 0.001; **p < 0.01; *p < 0.05. Standard errors in parentheses.

Note: Income groups follow OECD (2019) relative income thresholds anchored to the sample median: Poverty: < 50% of median; Below average: 50–100%; Above average: 100–200%; Affluence: ≥ 200% of median. Source: OECD (2019). Under Pressure: The Squeezed Middle Class. OECD Publishing.

Table S2: Average marginal comparisons for differences in meritocracy perception between income groups

Model	Comparison	Estimate	SE	z	p	95% CI
**Top-Bottom classification**						
Top-Bottom	Top 10% vs. Middle 50%	0.166	0.057	2.918	0.004**	[0.055, 0.278]
Top-Bottom	Top 10% vs. Bottom 40%	0.124	0.063	1.981	0.048*	[0.001, 0.247]
**OECD income classification**						
Median groups	Affluence vs. Above average	0.173	0.048	3.612	<0.001***	[0.079, 0.267]
Median groups	Affluence vs. Below average	0.096	0.051	1.904	0.057	[-0.003, 0.195]
Median groups	Above average vs. Below average	0.077	0.042	1.816	0.069	[-0.006, 0.159]

Note: * $p < 0.05$; ** $p < 0.01$; *** $p < 0.001$.

Comparisons are pairwise differences in predicted meritocracy perception between income groups, averaged over all other covariates.

Table S3: Ordinal Logistic Regression for socioeconomic status, subjective social status perception of meritocracy

	Effort			Talent		
	**Model 1**	**Model 2**	**Model 3**	**Model 4**	**Model 5**	**Model 6**
Income Decile	0.007	-0.110^{**}	0.006	0.011	-0.138^{***}	0.007
	(0.012)	(0.035)	(0.013)	(0.013)	(0.035)	(0.013)
Education (ref: Incomplete Primary or less)						
Primary & Lower secondary	-0.422^{**}	-0.397^{**}	-0.836^{*}	-0.373^{*}	-0.341^{*}	-1.308^{**}
	(0.151)	(0.151)	(0.399)	(0.151)	(0.151)	(0.401)
Upper secondary	-0.414^{***}	-0.397^{***}	-1.302^{***}	-0.473^{***}	-0.449^{***}	-1.223^{***}
	(0.118)	(0.118)	(0.302)	(0.120)	(0.120)	(0.310)
Short-cycle tertiary	-0.683^{***}	-0.677^{***}	-1.382^{***}	-0.626^{***}	-0.616^{***}	-1.806^{***}
	(0.138)	(0.138)	(0.383)	(0.139)	(0.139)	(0.388)
Tertiary or higher	-0.802^{***}	-0.835^{***}	-1.391^{***}	-0.735^{***}	-0.772^{***}	-2.115^{***}
	(0.139)	(0.139)	(0.351)	(0.141)	(0.141)	(0.359)
Subjective Social Status (SSS)	0.122^{***}	-0.019	-0.035	0.071^{**}	-0.108^{*}	-0.149^{*}
	(0.022)	(0.044)	(0.060)	(0.022)	(0.045)	(0.063)
Income × SSS		0.025^{***}			0.032^{***}	
		(0.007)			(0.007)	
Primary & Lower secondary × SSS			0.108			0.238^{**}
			(0.091)			(0.092)
Upper secondary × SSS			0.219^{**}			0.196^{**}
			(0.069)			(0.071)
Short-cycle tertiary × SSS			0.175^{*}			0.290^{***}
			(0.083)			(0.084)
Tertiary or higher × SSS			0.153^{*}			0.321^{***}
			(0.074)			(0.076)
Female (ref. = Male)	0.277^{***}	0.285^{***}	0.277^{***}	0.304^{***}	0.314^{***}	0.306^{***}
	(0.063)	(0.063)	(0.063)	(0.063)	(0.063)	(0.063)
Age	0.003	0.003	0.003	0.004	0.003	0.004
	(0.002)	(0.002)	(0.002)	(0.002)	(0.002)	(0.002)
Thresholds						
Threshold 1|2	-2.127^{***}	-2.754^{***}	-2.756^{***}	-2.518^{***}	-3.314^{***}	-3.398^{***}
	(0.190)	(0.257)	(0.292)	(0.193)	(0.260)	(0.301)
Threshold 2|3	0.249	-0.375	-0.376	-0.288	-1.079^{***}	-1.162^{***}
	(0.184)	(0.251)	(0.288)	(0.184)	(0.253)	(0.295)
Threshold 3|4	1.176^{***}	0.555^{*}	0.554	0.706^{***}	-0.080	-0.164
	(0.185)	(0.251)	(0.288)	(0.184)	(0.252)	(0.294)
Threshold 4|5	3.359^{***}	2.741^{***}	2.739^{***}	3.184^{***}	2.405^{***}	2.319^{***}
	(0.197)	(0.259)	(0.295)	(0.197)	(0.259)	(0.300)
Num. obs.	3488	3488	3488	3488	3488	3488
AIC	9.745.393	9.734.296	9.742.555	9.758.188	9.739.389	9.746.000
BIC	9.819.350	9.814.416	9.841.165	9.832.145	9.819.510	9.844.609
Log Likelihood	-4.860.696	-4.854.148	-4.855.277	-4.867.094	-4.856.695	-4.857.000

$^{***}p < 0.001$; $^{**}p < 0.01$; $^{*}p < 0.05$. Standard errors in parentheses.

Table S4. Tests of the Proportional Odds (Parallel Lines) Assumption

Predictor	Df	LRT	p-value
**Effort**			
Income	3	15.68	0.001
Education	12	47.91	<.001
Subjective Social Status	3	9.04	0.029
Sex	3	2.18	0.535
Age	3	9.69	0.021
**Talent**			
Income	3	29.31	<.001
Education	12	48.47	<.001
Subjective Social Status	3	16.41	<.001
Sex	3	5.44	0.142
Age	3	10.08	0.018

Note. LRT = Likelihood Ratio Test statistic. Partial proportional odds models were estimated for predictors where the assumption was violated ($p < .05$). Sex retained the proportional odds constraint in both models.

Table S5: Linear Regression for socioeconomic status, subjective social status perception of meritocracy

	**Model 1**	**Model 2**
Income Decile	-0.001	-0.002
	(0.006)	(0.006)
Subjective Social Status (SSS)	0.048^{***}	0.036^{**}
	(0.011)	(0.013)
Universitary (ref.= Short-cycle or lower)	-0.158^{***}	-0.424^{**}
	(0.043)	(0.138)
Universitary × SSS		0.053^{*}
		(0.026)
Female (ref.= Male)	0.167^{***}	0.166^{***}
	(0.033)	(0.033)
Age	0.004^{***}	0.004^{***}
	(0.001)	(0.001)
R^2	0.019	0.020
Adj. R^2	0.017	0.018
Num. obs.	3488	3488

$^{***}p < 0.001$; $^{**}p < 0.01$; $^{*}p < 0.05$. Standard errors in parentheses.

Table S6: Linear Regression for socioeconomic status, subjective social status perception of meritocracy

	**Model 1**	**Model 2**	**Model 3**
Income Decile	0.005	-0.066^{***}	0.003
	(0.007)	(0.018)	(0.007)
Education (ref: Incomplete Primary or less)			
Primary & Lower secondary	-0.191^{*}	-0.180^{*}	-0.573^{**}
	(0.078)	(0.077)	(0.201)
Upper secondary	-0.231^{***}	-0.220^{***}	-0.701^{***}
	(0.062)	(0.062)	(0.157)
Short-cycle tertiary	-0.362^{***}	-0.354^{***}	-0.886^{***}
	(0.072)	(0.072)	(0.199)
Tertiary or higher	-0.422^{***}	-0.442^{***}	-0.977^{***}
	(0.073)	(0.073)	(0.183)
Subjective Social Status (SSS)	0.054^{***}	-0.031	-0.052
	(0.011)	(0.023)	(0.031)
Income × SSS		0.015^{***}	
		(0.004)	
Primary & Lower secondary × SSS			0.097^{*}
			(0.046)
Upper secondary × SSS			0.118^{***}
			(0.036)
Short-cycle tertiary × SSS			0.130^{**}
			(0.043)
Tertiary or higher × SSS			0.133^{***}
			(0.038)
Female (ref.= Male)	0.169^{***}	0.175^{***}	0.169^{***}
	(0.033)	(0.033)	(0.033)
Age	0.002^{*}	0.002	0.002^{*}
	(0.001)	(0.001)	(0.001)
Indigenous (ref.= Non-indigenous)	-0.038	-0.035	-0.048
	(0.052)	(0.052)	(0.052)
Immigrant (ref.= Native)	0.396^{***}	0.376^{***}	0.392^{***}
	(0.087)	(0.087)	(0.087)
R^2	0.032	0.037	0.036
Adj. R^2	0.029	0.034	0.032
Num. obs.	3461	3461	3461

$^{***}p < 0.001$; $^{**}p < 0.01$; $^{*}p < 0.05$. Standard errors in parentheses.

Table S7: Descriptive Statistics for Study Variables

Variable	N	Mean	SD	Min	Max
Perceived meritocracy	3488	2.8	0.98	1	5
Subjective Social Status	3488	4.4	1.6	0	10
Household Income	3488	5.5	2.9	1	10
Education	3488				
... Incomplete Primary or lower		12%			
... Primary & Lower secondary		10%			
... Upper secondary		43%			
... Short-cycle tertiary		16%			
... Tertiary or higher		19%			
Gender	3488				
... Female		62%			
... Male		38%			
Age	3488	47	15	18	90

References

OECD. (2019). *Under pressure: The squeezed middle class*. OECD Publishing. https://doi.org/10.1787/689afed1-en
